# Gut Microbiome and Metabonomic Profile Predict Early Remission to Anti-Integrin Therapy in Patients with Moderate to Severe Ulcerative Colitis

**DOI:** 10.1128/spectrum.01457-23

**Published:** 2023-05-18

**Authors:** Jie Liu, Huaying Fang, Na Hong, Chaolan Lv, Qihua Zhu, Yinping Feng, Bo Wang, Jiashuang Tian, Yue Yu

**Affiliations:** a Department of Gastroenterology, The First Affiliated Hospital of USTC, Division of Life Sciences and Medicine, University of Science and Technology of China, Hefei, Anhui, People’s Republic of China; b Endoscopy Center Department, The First Affiliated Hospital of USTC, Division of Life Sciences and Medicine, University of Science and Technology of China, Hefei, Anhui, People’s Republic of China; c Department of Nursing, The First Affiliated Hospital of USTC, Division of Life Sciences and Medicine, University of Science and Technology of China, Hefei, Anhui, People’s Republic of China; Jilin University

**Keywords:** anti-integrin therapy, metabonomics, microbiology, ulcerative colitis

## Abstract

Patients with ulcerative colitis (UC) have low response rates to anti-integrin medications, necessitating the identification of noninvasive biomarkers for predicting remission to anti-integrin therapy. In this study, patients with moderate to severe UC commencing anti-integrin therapy (*n* = 29), inactive to mild UC patients (*n* = 13), and healthy controls (*n* = 11) were selected. Besides clinical evaluation, fecal samples were collected at baseline and week 14 from moderate to severe UC patients. The clinical remission was defined based on the Mayo score. Fecal samples were assessed with 16S rRNA gene sequencing, liquid chromatography-tandem mass spectrometry, and gas chromatography-mass spectrometry (GC-MS). We identified that *Verrucomicrobiota* was significantly more abundant in the remission group (*P* < 0.001) than that of nonremission group at phylum level for patients commencing vedolizumab. GC-MS analysis revealed that the concentrations of butyric acid (*P* = 0.024) and isobutyric acid (*P* = 0.042) were significantly higher in the remission group compared to the nonremission group at baseline. Finally, the combination of *Verrucomicrobiota*, butyric acid, and isobutyric acid improved the diagnosis of early remission to anti-integrin therapy (area under the concentration-time curve = 0.961). We identified significantly higher phylum level diversity of *Verrucomicrobiota* in remission than the nonremission groups at baseline. Notably, the combination of gut microbiome and metabonomic profiles improved the diagnosis of early remission to anti-integrin therapy.

**IMPORTANCE** It is reported that patients with ulcerative colitis (UC) have low response rates to anti-integrin medications in the latest VARSITY study. Therefore, our primary goals were to discover differences in the gut microbiome and metabonomics patterns between early remission and nonremission patients and to explore the diagnostic value in predicting clinical remission to anti-integrin therapy accurately. In this study, we found that *Verrucomicrobiota* was significantly more abundant in the remission group (*P* < 0.001) than that of nonremission group at phylum level for patients commencing vedolizumab. Gas chromatography-mass spectrometry analysis revealed that the concentrations of butyric acid (*P* = 0.024) and isobutyric acid (*P* = 0.042) were significantly higher in the remission group compared with the nonremission group at baseline. Notably, the combination of *Verrucomicrobiota*, butyric acid, and isobutyric acid improved the diagnosis of early remission to anti-integrin therapy (area under the concentration-time curve = 0.961).

## INTRODUCTION

Ulcerative colitis (UC), an inflammatory bowel disease (IBD), is characterized by chronic, idiopathic, relapsing, and remitting inflammation. UC typically begins in the rectum and progresses proximally across a portion of the colon or the entire colon, affecting the distal colonic mucosa and submucosa (1). UC is more prevalent in industrialized areas, resulting in an estimated prevalence of 286 per 100,000 adult Americans (2, 3). Several novel therapeutic targets have been proven helpful in treating IBD (4), whereas tumor necrosis factor (TNF) antagonists remain the cornerstone of treatment for moderately to highly active IBD, with approximately 70% primary response rates in placebo-controlled trials (5, 6). However, the loss of responsiveness and systemic nature of anti-TNF-α medicines have necessitated the exploration of alternate signaling pathway inhibitors (6).

Vedolizumab, a gut-selective antilymphocyte trafficking agent, is approved for treating IBD. Vedolizumab is the first biological drug to selectively target α4β7 gastrointestinal integrin receptors, thereby downregulating lymphocyte trafficking into colonic tissue (7). Initial controlled studies in the real world demonstrated that vedolizumab has a more significant or comparable effect to anti-TNF-α therapies (8–10). Another study found that vedolizumab was equally effective to anti-TNF-α in controlling inflammatory activity but with better safety profiles of patients (11). Vedolizumab is the first biological therapy for UC or suggested for patients who fail anti-TNF medications (12). However, the latest VARSITY study (13) reported remission in week 14 in only 34% of UC patients (50/147). Moreover, UC patients who started vedolizumab early had a higher chance of remission at week 52 than delayed responders. Therefore, there is a growing interest in identifying alternative, noninvasive biomarkers to predict early responsiveness to anti-integrin therapy (14).

Several promising biomarkers, including oncostatin M, Il13RA2, αE, and TREM-1, have been validated for predicting response to biological therapies in treating IBD; however, they are not yet available in clinical practice (15–18). A prospective cohort study (19) predicted response to anti-integrin therapy by employing fecal microbiota of Crohn’s disease (CD) patients at baseline. The study observed an abundance of Roseburia inulinivorans and a *Burkholderiales* sp. in CD patients attaining early remission at week 14 than in the nonremission group before anti-integrin biologic therapy. Another study that analyzed the metabolic patterns of serum, urine, and feces among CD patients found that noninvasive biomarkers are likely to play a pivotal role in predicting remission to anti-TNF-α therapies (20).

Several studies have revealed that specific gut microbiome and metabonomics profiles are related to the primary response to biological agents in CD. However, the association between these profiles and remission in UC patients starting vedolizumab is largely unknown. Therefore, this pilot study sought (i) to identify the differences in gut microbiome and metabonomics patterns among cases of inactive to mild UC and moderate to severe UC and among healthy controls; (ii) to compare gut microbiota and metabonomics patterns between early-remission and nonremission patients who received anti-integrin therapy; (iii) to explore metabolic and microbial predictors to accurately predict the remission to anti-integrin therapy in moderate to severe UC patients; and (iv) to identify the longitudinal trajectory in the microbiome with maintenance treatment.

## RESULTS

### Demographics data of enrolled participants.

A total of 13 inactive to mild UC patients, 29 moderate to severe UC patients and 11 healthy controls, were included. The average ages of the healthy controls, the inactive to mild UC patients, and the moderate to severe UC patients were 36.09 ± 7.57 years, 46.00 ± 15.72 years, and 42.76 ± 15.67 years, respectively. The demographics of the UC patients were statistically insignificant compared to controls (see Table S1 in the supplemental material). There was also an insignificant difference in dietary intakes between UC patients and healthy controls based on food frequency questionnaires (FFQs) (see Table S2).

### Clinical data.

The baseline Mayo scores for inactive to mild and moderate to severe UC patients were 3.77 ± 0.83 and 8.24 ± 1.46; C-reactive protein (CRP), 8.97 ± 4.19 mg/L and 18.09 ± 5.23 mg/L; and fecal calprotectin (FC), 54.46 ± 26.73 μg/g and 142.10 ± 36.01 μg/g, respectively. A cohort of 29 patients with moderate to severe UC who received vedolizumab therapy was monitored for 14 weeks. At week 14, 41.38% (12/29) of the patients achieved remission based on the Mayo score and endoscopy outcomes. The differences in clinical and biochemical characteristics of inactive to mild and moderate to severe UC patients are shown in Table S3 in the supplemental material. The differences in clinical and biochemical characteristics between the vedolizumab remission and nonremission groups are presented in Table 1. The typical endoscopic appearances of moderate to severe UC patients before and after vedolizumab treatment between remission and nonremission after 14 weeks of follow-up are shown in Fig. S2.

**TABLE 1:** Summary of clinical and biochemical characteristics between vedolizumab responders and nonresponders among moderate to severe UC patients

Clinical parameter	Moderate to severe UC (*n* = 29)^*a*^		*t*/χ^2^	*P*
	Remission (*n* = 12)	Nonremission (*n* = 17)		
General				
Age (yrs)	38.92 ± 16.03	45.47 ± 15.30	−1.114	0.275
Female (n)	3	7	0.815	0.449
Duration (mo)	68.42 ± 17.63	64.59 ± 18.79	0.934	0.359
Body mass index (kg/m^2^)	21.31 ± 1.86	22.04 ± 2.60	−0.832	0.413
Hemoglobin (g/L)	114.17 ± 20.85	120.88 ± 22.84	−0.808	0.426
White cell count (×10^9^/L)	6.35 ± 1.88	7.19 ± 2.37	−1.015	0.319
Platelet count (×10^9^/L)	225.33 ± 83.45	259.53 ± 76.07	−1.146	0.262
ESR (mm/h)	36.52 ± 10.86	28.42 ± 12.28	1.589	0.124
Inflammatory biomarkers				
C-reactive protein (mg/L)	19.72 ± 5.77	17.50 ± 5.12	−0.719	0.478
Calprotectin (μg/g)	164.75 ± 30.66	126.12 ± 42.05	0.747	0.461
Liver function				
Bilirubin (μM)	10.21 ± 3.36	13.50 ± 4.21	−1.788	0.085
Alkaline phosphatase (IU/L)	81.67 ± 29.71	83.00 ± 26.19	−0.075	0.941
Alanine aminotransferase (IU/L)	11.34 ± 3.65	19.79 ± 4.62	−1.544	0.134
Aspartate aminotransferase (IU/L)	16.05 ± 4.78	20.75 ± 5.41	1.587	0.124
Albumin (g/L)	38.17 ± 5.08	37.29 ± 5.49	0.442	0.662
Renal function				
Creatinine (μM)	62.08 ± 10.78	65.53 ± 14.61	−0.693	0.494
Total Mayo score	8.17 ± 1.19	8.29 ± 1.65	−0.228	0.281
Endoscopic Mayo score	2.17 ± 0.39	2.12 ± 0.33	0.365	0.718
Disease extent				
Pancolitis (*n*)	6	8	0.024	0.876
Left-sided colitis (*n*)	6	9		
Concomitant medications				
5-ASA (*n*)^*b*^	8	13		0.683
Prior anti-TNF failure				
Infliximab (*n*)	5	7	0.001	0.979
Adalimumab (*n*)	2	3		1.000

a Values are expressed as means ± the standard deviations or numbers of subjects (*n*).

b 5-ASA, 5-aminosalicylic acid. The difference in 5-ASA and adalimumab between the two groups was calculated by using a Fisher exact test.

### Microbial profiles between healthy controls and UC patients.

**FIG 1:**
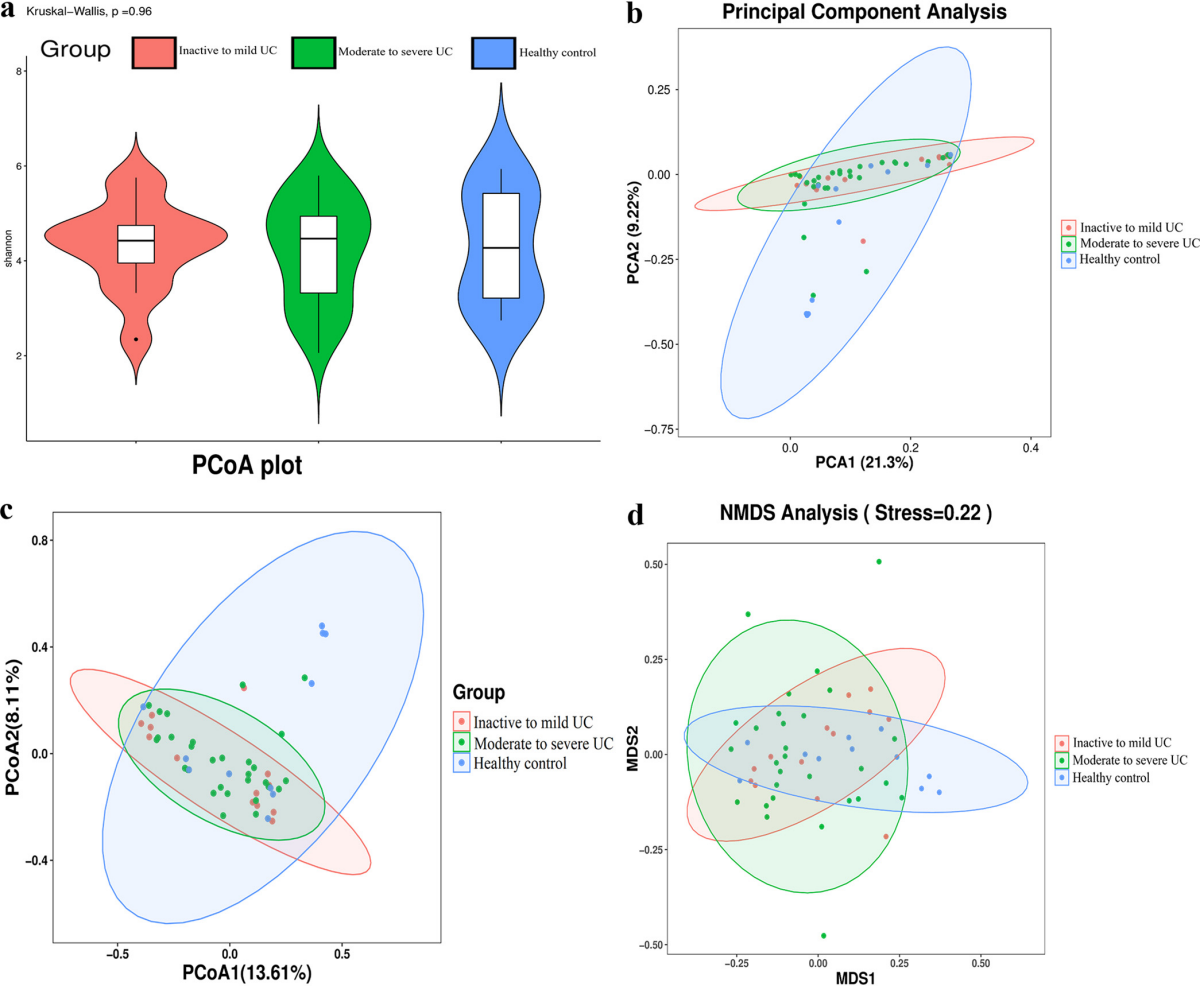
Gut microbiota between healthy controls and UC patients. (a) Shannon’s diversity index showing an insignificant difference (Kruskal-Wallis test, *P* = 0.96) in α-diversity between the three groups. (b to d) PCA (b), PCoA (c), and NMDS (d) analyses (stress = 0.22) showed an insignificant difference in β-diversity using between the three groups.

**FIG 2:**
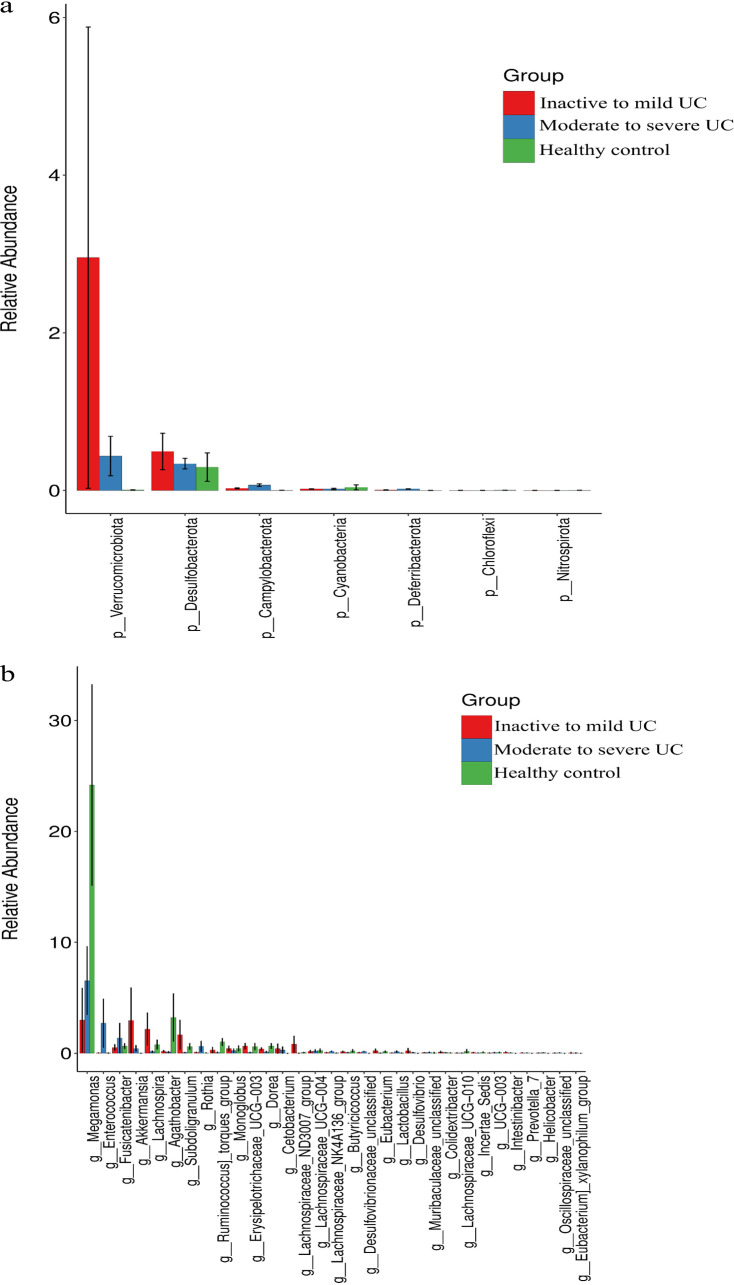
Gut microbiota analysis at phylum and genus levels between healthy controls, inactive to mild UC patients, and moderate to severe UC patients. (a) The relative abundance of *Campylobacterota* at the phylum level in healthy controls was significantly lower than in inactive to mild UC patients (*P* < 0.001) and moderate to severe UC patients (*P* < 0.001). (b) The relative abundance of *Megamonas* in healthy controls was significantly higher than in inactive to mild UC patients and moderate to severe UC patients (*P* < 0.001).

An insignificant difference in α-diversity was detected by comparing Shannon’s diversity index (Kruskal-Wallis test, *P* = 0.960) between the three groups (Fig. 1a). There was also an insignificant difference found in β-diversity through principal component analysis (PCA) (Fig. 1b), principal coordinate analysis (PCoA) (Fig. 1c), and nonmetric multidimensional scaling (NMDS) analysis (stress = 0.220) (Fig. 1d) between the three groups. The taxonomy community analysis revealed that there was no difference in the levels of *Firmicutes* and *Bacteroidota* at the phylum level between healthy controls, inactive to mild UC patients, and moderate to severe UC patients. However, the levels of *Verrucomicrobiota* at the phylum level in inactive to mild UC patients were significantly higher than those of healthy controls (*P* < 0.001) and moderate to severe UC patients (*P* < 0.001). Meanwhile, the levels of *Campylobacterota* at the phylum level in healthy controls were significantly lower than those of inactive to mild UC patients (*P* < 0.001) and moderate to severe UC patients (*P* < 0.001) (Fig. 2a). The genus-level relative abundance of *Megamonas* in healthy controls was significantly higher than in inactive to mild and moderate to severe UC patients (*P* < 0.001). In addition, the relative abundance of *Enterococcus* in moderate to severe UC patients was significantly higher than that in inactive to mild UC patients and in healthy controls (*P* < 0.001) (Fig. 2b).

### Microbial profiles between remission and nonremission groups.

**FIG 3:**
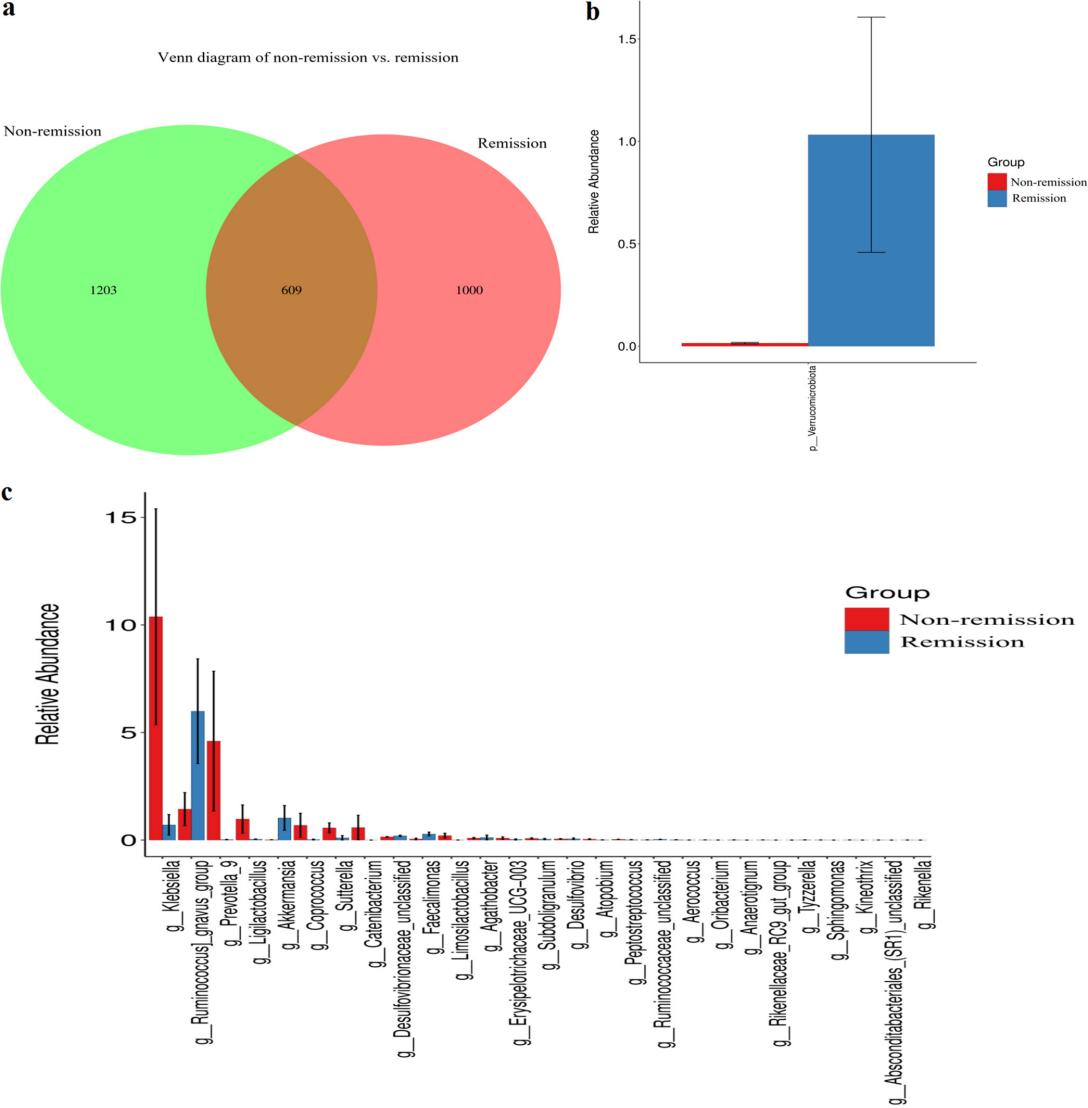
Gut microbiota between remission and nonremission groups. (a) Venn diagram showing common taxa. (b and c) Taxonomy community difference analyses at the phylum level (b) and at the genus level (c) between remission and nonremission groups.

A Venn diagram illustrating the shared core and unique intestinal bacterial genera between remission and nonremission patients of moderate to severe UC patients who underwent anti-integrin therapy was prepared (Fig. 3a). Our analysis revealed that 21.66% (609/2,812) of the genera were shared between the two groups, while 42.78% (1,203/2,812) were unique to nonremission patients and 35.56% (1,000/2,812) were unique to remission patients. In addition, taxonomy community analysis was performed and, at the phylum level, our results indicated that *Verrucomicrobiota* was significantly more abundant in the remission group compared to nonremission patients (*P* < 0.001) (Fig. 3b). At the genus level, *Ruminococcus* (*P* = 0.046) and *Akkermansia* (*P* < 0.001) in the remission patients were markedly more abundant than those of nonremission patients (Fig. 3c). Finally, we performed functional prediction analysis to investigate the difference in the direction of modification for pathways between those attaining remission and those not. Our research revealed potential pathways, including the superpathway of l-threonine biosynthesis, pyruvate fermentation to propanoate, *cis*-vaccenate biosynthesis, and others (see Fig. S3).

### Untargeted fecal metabolic profiles of healthy controls and UC patients.

**FIG 4:**
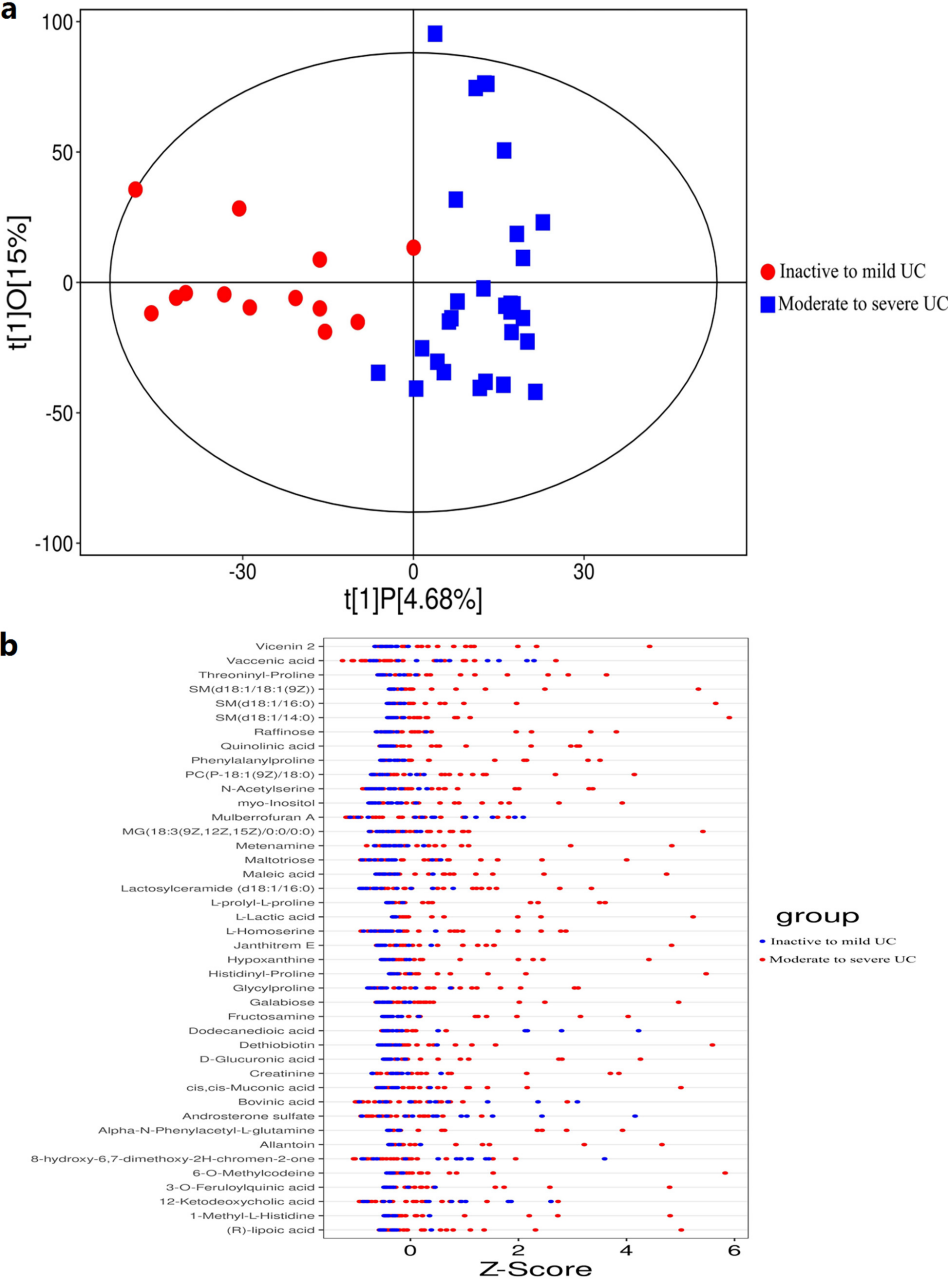
Differences in untargeted fecal metabolic profiles between inactive to mild UC patients and moderate to severe UC patients. (a) OPLS-DA models showed that the samples of moderate to severe UC patients formed a cluster of samples that is distinguished from the samples of inactive to mild UC patients. (b) Untargeted fecal metabolic profiling revealed a significant difference between inactive to mild UC patients and moderate to severe UC patients, including vicenin 2, vaccenic acid, threoninyl-proline, and others.

**FIG 5:**
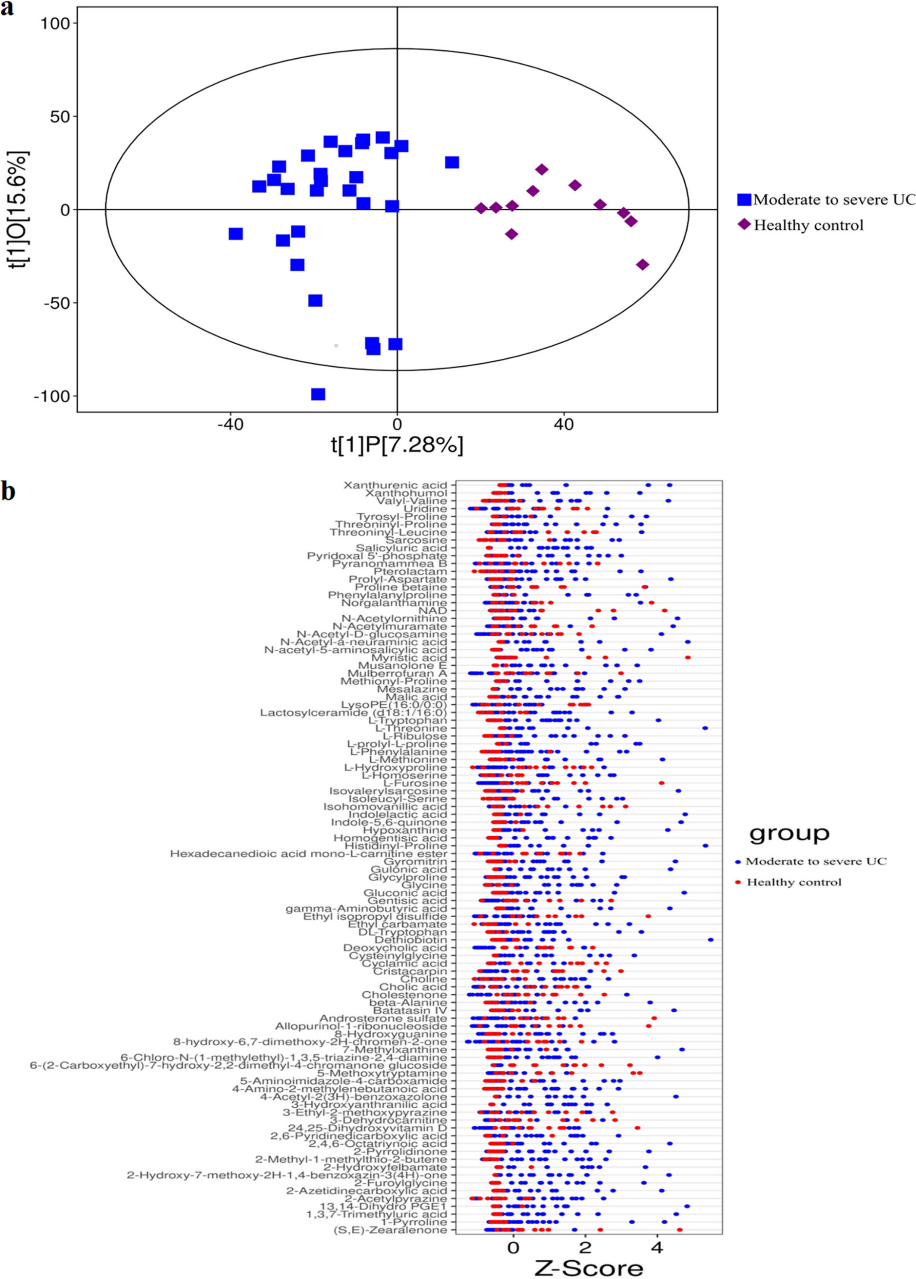
Differences in untargeted fecal metabolic profiles between healthy controls and moderate to severe UC patients. (a) OPLS-DA models showed that the samples of patients with moderate to severe UC formed a cluster of samples that is distinguished from the samples of health controls. (b) Untargeted fecal metabolic profile analysis showed a significant difference between patients with moderate to severe UC and healthy controls, including xanthurenic acid, xanthohumol, valyl-valine, and others.

Robust OPLS-DA models demonstrated that samples of moderate to severe UC patients formed a cluster distinct from the samples of inactive to mild UC patients (Fig. 4a). Analysis of untargeted fecal metabolic profiles revealed a substantial difference between moderate to severe and inactive to mild UC patient profiles, including vicenin 2, vaccenic acid, threoninyl-proline, and others (Fig. 4b). Similarly, robust OPLS-DA (orthogonal projections to latent structures discriminant analysis) models demonstrated a cluster difference between moderate to severe UC patient samples and healthy controls (Fig. 5a). Analysis of untargeted fecal metabolic profiles revealed a significant difference between moderate to severe UC patients and healthy controls, including xanthurenic acid, xanthohumol, valyl-valine, and others (Fig. 5b).

### Untargeted fecal metabolic profiles between remission and nonremission groups.

**FIG 6:**
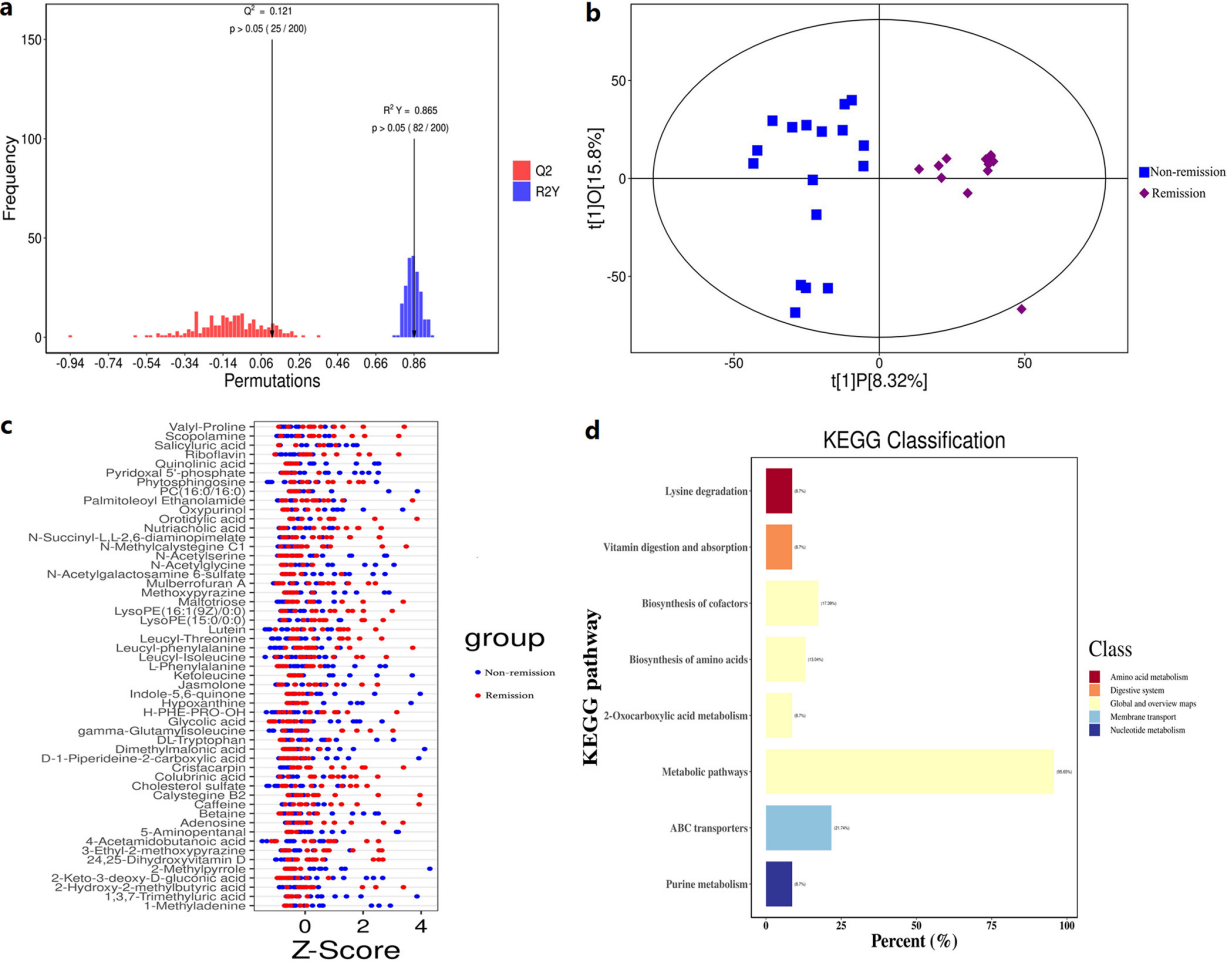
Differences in untargeted fecal metabolic profiles between remission and nonremission patients. (a) OPLS-DA models were validated by the statistical Q^2^Y, and permutation testing showed significant metabolic differences between remission and nonremission groups. (b) OPLS-DA revealed a cluster difference between remission and nonremission groups. (c) Untargeted fecal metabolic profiling showed a significant difference between remission and nonremission groups. (d) KEGG enrichment analysis showed an association of the differential abundance score between the two groups with pathways, including lysine degradation, vitamin digestion and absorption, biosynthesis of cofactor pathways, and others.

Robust OPLS-DA models for fecal metabolites revealed significant metabolic differences between remission and nonremission patients of moderate to severe UC patients who underwent anti-integrin therapy, as validated by statistical Q^2^Y and permutation testing (Fig. 6a). OPLS-DA showed that samples of the remission group formed a distinct cluster from samples of nonremission groups of UC patients (Fig. 6b). Analysis of untargeted fecal metabolic profiles noted a significant difference between the two groups, including proline, scopolamine, salicyluric acid, riboflavin, quinolinic acid, and others (Fig. 6c). KEGG enrichment analysis revealed that the differential abundance score between the two groups is associated with lysine degradation, vitamin digestion and absorption, biosynthesis of cofactors, and other similar pathways (Fig. 6d).

### SCFA differences between remission and nonremission groups.

**FIG 7:**
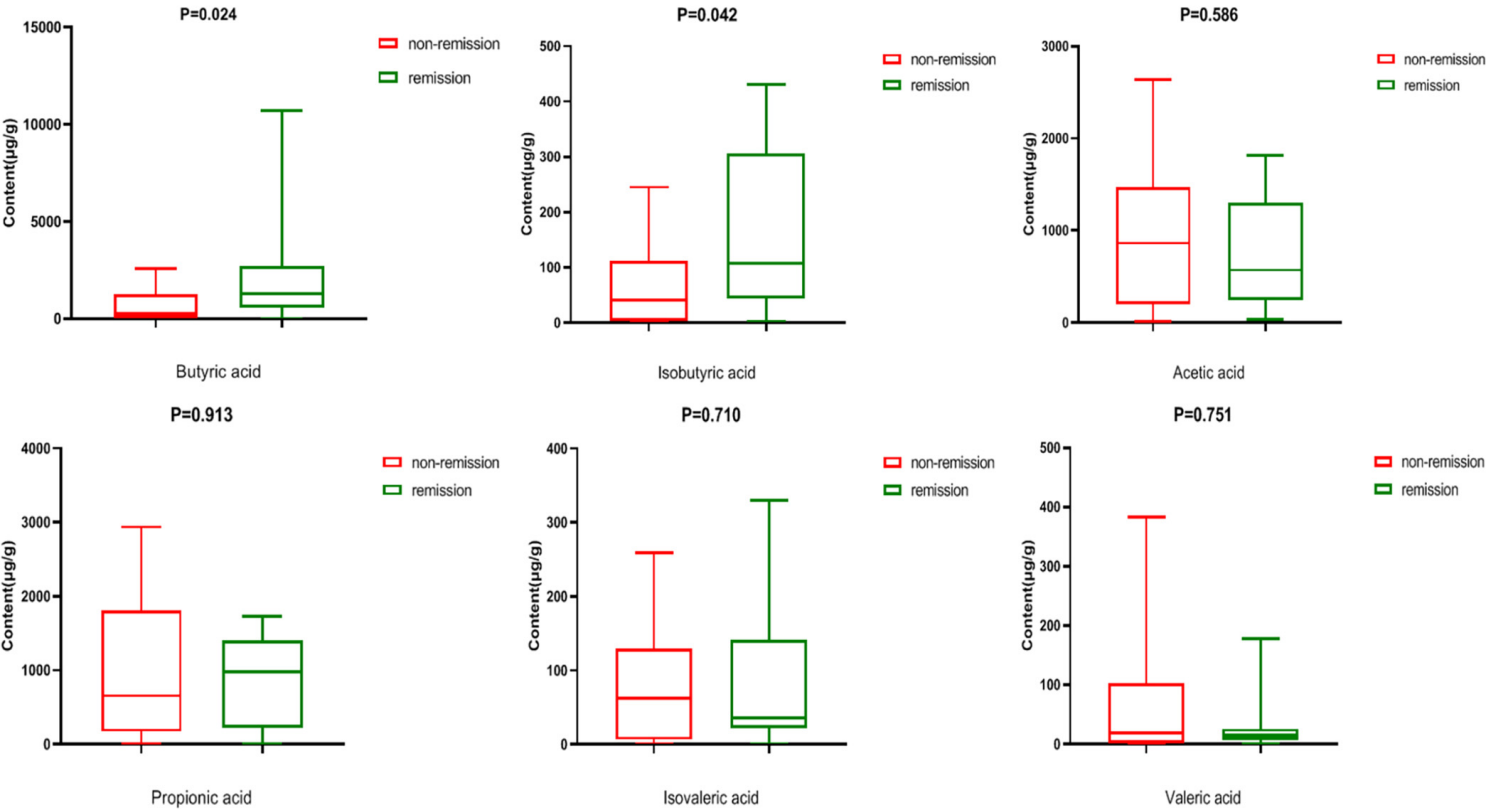
Differences in SCFAs between remission and nonremission groups in moderate to severe UC patients starting vedolizumab treatment. GC-MS analysis showed significantly higher butyric acid (*P* = 0.024) and isobutyric acid (*P* = 0.042) levels in moderate to severe UC patients who attained remission at week 14 after anti-integrin therapy than in those who did not achieve remission at baseline.

Gas chromatography-mass spectrometry (GC-MS) was conducted to assess the short-chain fatty acid (SCFA) content in UC patients who underwent anti-integrin therapy at baseline. Significantly higher levels of butyric acid (*P* = 0.024) and isobutyric acid (*P* = 0.042) were observed in moderate to severe UC patients who attained remission after anti-integrin therapy at week 14 compared to those who did not achieve remission at baseline. However, no differences were observed in other SCFAs between the two groups (Fig. 7). Spearman’s correlation analysis revealed significant associations between disease severity score (Mayo score) and propionic acid (*P* = 0.019), butyric acid (*P* = 0.028), and valeric acid (*P* = 0.039) in moderate to severe UC patients (see Fig. S4 and Table S4).

### Metabolic and microbial predictors of anti-integrin response in UC patients.

**FIG 8:**
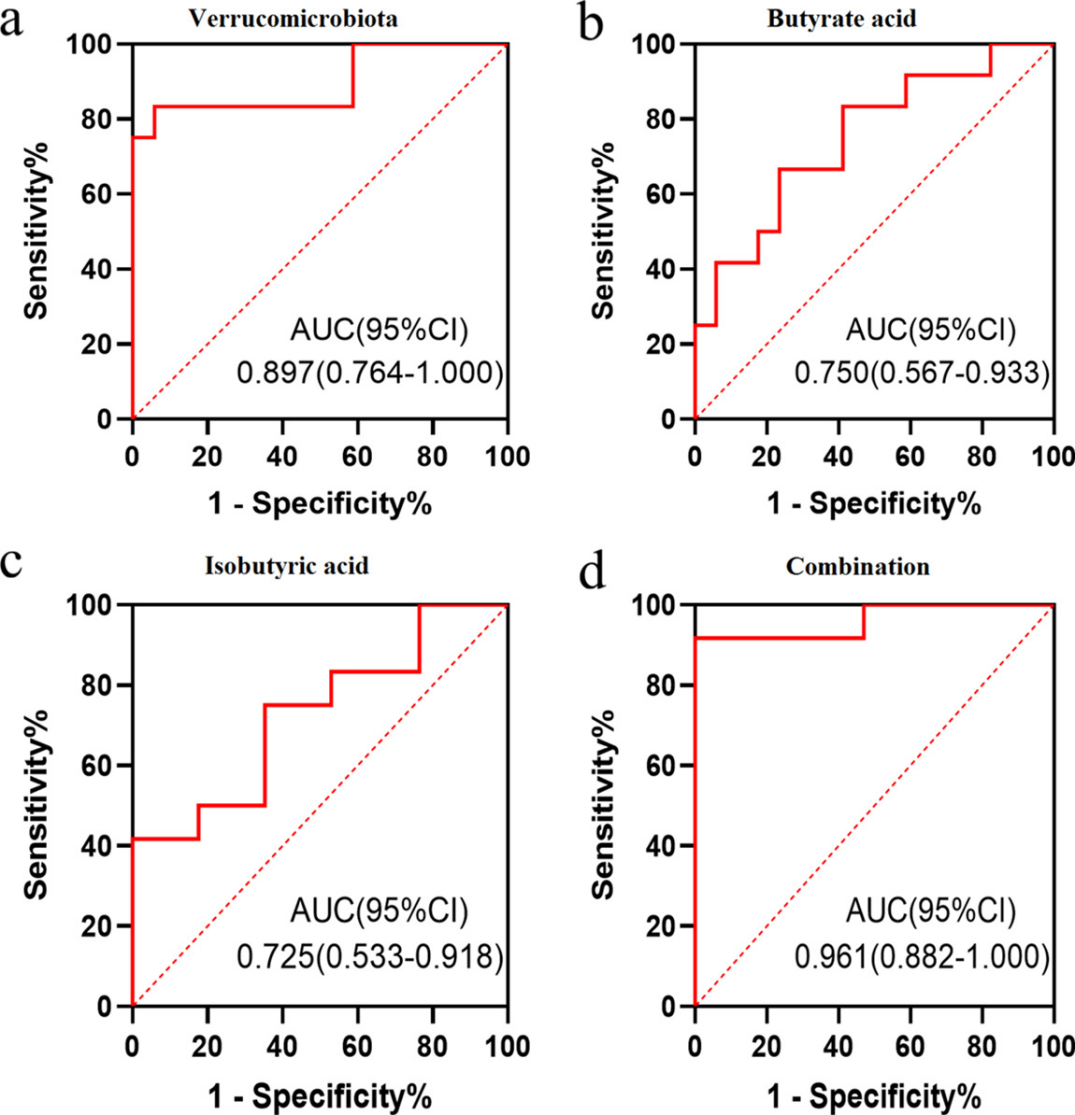
Diagnostic value utilizing gut microbiome and metabonomics pattern in predicting clinical remission. (a) An AUROC, area under the receiver operating characteristic curve of 0.897 with 95% CI (0.764 to 1.000) was established using *Verrucomicrobiota* at the phylum level. (b) Butyric acid with a moderate discriminating ability with an AUROC of 0.750 with 95% CI (0.567 to 0.933). (c) Isobutyric acid with a moderate discriminating ability with an AUROC of 0.725 with 95% CI (0.533 to 0.918). (d) Combining the fecal microbiota marker with butyrate and isobutyric acid gave the most potent discriminating ability, with an AUROC of 0.96 (1.082 to 1.00).

*Verrucomicrobiota* at the phylum level showed high discriminating ability (area under the concentration-time curve from 0 h to infinity [AUC] = 0.897; 95% confidence interval [95% CI] = 0.764 to 1.000) in UC patients undergoing anti-integrin therapy, while butyric acid (AUC = 0.750; 95% CI = 0.567 to 0.933) and isobutyric acid (AUC = 0.725; 95% CI = 0.533 to 0.918) showed moderate discriminating abilities. Combining the fecal microbiota marker with butyrate and isobutyric acid resulted in the highest diagnostic value, with an AUC of 0.961 (95% CI = 0.882 to 1.000), indicating a model with a superior predictive ability for predicting anti-integrin therapy response (Fig. 8). Table 2 displays the cutoff value, sensitivity, and specificity of each parameter for predicting the response to vedolizumab.

**TABLE 2:** Receiver operating characteristic association statistics for predicting response to vedolizumab

Substrate	AUC^*a*^	95% CI	Cutoff value	%		*P*
				Sensitivity	Specificity	
*Verrucomicrobiota*	0.897	0.764–1.000	0.04	83.3	94.1	<0.001
Butyrate	0.750	0.567–0.933	1125.7	66.7	76.5	0.024
Isobutyrate	0.725	0.533–0.918	250.69	41.7	100.0	0.042
*Verrucomicrobiota *+ butyrate + isobutyrate	0.961	0.882–1.000	0.4079	91.7	100.0	<0.001

a AUC, area under the curve; 95% CI, 95% confidence interval.

### Longitudinal trajectory of fecal microbiota before and after anti-integrin therapy.

**FIG 9:**
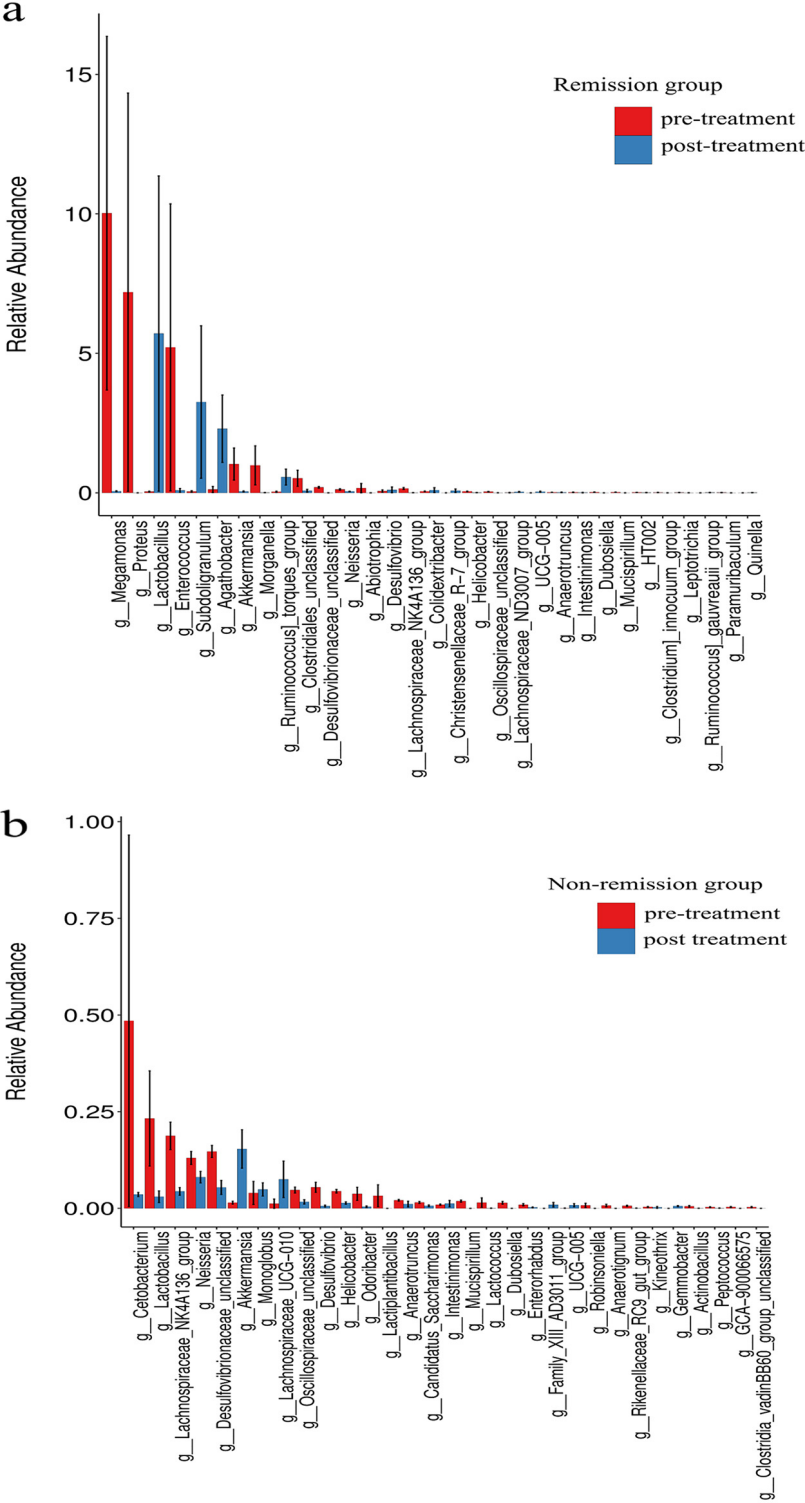
Longitudinal changes of microbial profiles between pre- and post-anti-integrin therapy. (a) An increase in the genus *Lactobacillus* probiotics (*P* = 0.021) was detected for UC patients who attained remission at week 14. (b) Nonremission patients showed a decrease in *Cetobacterium* (*P* < 0.001) and *Lactobacillus* (*P* < 0.001) and an increase in *Akkermansia* (*P* < 0.001) at the genus level.

A comparison of pre- and post-anti-integrin treatment samples from UC patients undergoing anti-integrin therapy (including remission and nonremission patients) revealed a decreased abundance of *Megamonas* (*P* = 0.025) and an increased abundance of *Lactobacillus* probiotics (*P* = 0.021) at the genus level in remission patients at week 14 (Fig. 9a). However, nonremission patients showed a decreased abundance of *Cetobacterium* (*P* < 0.001) and *Lactobacillus* (*P* < 0.001) and an increased abundance of *Akkermansia* (*P* < 0.001) at the genus level at week 14 (Fig. 9b).

## DISCUSSION

In the VARSITY study (13), only 34% of UC patients commencing vedolizumab achieved remission at week 14. Furthermore, patients who attained early remission were more likely to attain remission at week 52 than did delayed responders. Therefore, improving the discrimination of early remission to anti-integrin therapy in UC patients is an urgent concern. In the present study, we found that 41.38% of UC patients achieved remission at week 14, which is similar to the VARSITY study (13). Meanwhile, this study observed a difference in the microbial and metabolic signature between UC patients and healthy controls. Specifically, *Verrucomicrobiota* was significantly more abundant at the phylum level in patients initiating vedolizumab with remission than in nonremission patients. GC-MS analysis also showed that butyric acid and isobutyric acid concentrations were significantly higher in patients with remission than in nonremission patients at baseline. Moreover, the combination of *Verrucomicrobiota*, butyric acid, and isobutyric acid improves the diagnosis of early remission to anti-integrin therapy. In addition, a longitudinal trajectory in fecal microbiota was observed before and after anti-integrin therapy in both remission and nonremission groups.

### Dysbiosis of intestinal flora in UC compared to healthy control.

The results of many studies comparing the gut microbes between UC patients and healthy controls are varied or even contradictory. Previous studies indicated a decline in fecal microbiota diversity (21–24), while our study found insignificant differences in α- and β-diversity between healthy controls, inactive to mild UC, and moderate to severe UC patients, which might be owing to a small sample size employed in this study. A recent study found a significantly low phylum level abundance of *Bacteroidetes* and a significantly higher order-level abundance of *Actinomycetales* and family-level abundance of *Leptotrichiaceae* in UC patients compared to healthy controls (21). Another study found sulfate-reducing bacteria to be more abundant in UC patients than in healthy controls, possibly due to toxic sulfide production (25). The present study found that the phylum-level abundance of *Campylobacterota* was markedly higher in inactive to mild and moderate to severe UC patients than in healthy controls, showing consistency with Liu et al. (26).

### Gut metabonomic profile in UC patients compared to healthy controls.

Microbially derived metabolites serve as signaling molecules known to regulate immunological homeostasis and play a key role in development of IBD. A cohort study identified markedly differences in intestinal metabolite patterns between healthy controls and active IBD patients (27, 28). Julian and colleagues (29) found decreased fecal acetic acid and butyric acid in UC patients coompared to healthy controls by employing ^1^H nuclear magnetic resonance spectroscopy. Similarly, GC-MS analysis detected lower fecal propionic acid and acetic acid levels in UC patients than in healthy controls (22). Moreover, IBD has been associated with altered small molecules such as SCFAs, amino acids, and bile acids (30–32). Here, however, untargeted fecal metabolic profiling revealed significant differences between moderate to severe UC patients and healthy controls, including compounds such as xanthurenic acid, xanthohumol, valyl-valine, and others.

### Distinct microbial profiles between remission and nonremission patients.

Magnusson et al. (33) reported a higher abundance of Faecalibacterium prausnitzii and a lower dysbiosis index in anti-TNF therapy responders compared to nonresponders in UC patients. Conversely, Vatn et al. (34) found insignificant differences in bacterial markers between responders and nonresponders. Estevinho et al. (35) observed a lower abundance of Escherichia coli and a higher abundance of SCFA-producing bacteria in anti-TNF therapy responders than in nonresponders. Likewise, a prior study (36) demonstrated that anti-TNF therapy nonresponders exhibited a reduction in biodiversity and SCFA-producing bacteria. Our study indicated a higher phylum-level abundance of *Verrucomicrobiota* in patients with remission than in the nonremission group. It has been reported that *Verrucomicrobiota* can be easily detected by 16S rRNA sequencing and is one of the phyla present in the human gut. Akkermansia muciniphila has been demonstrated as the only cultivated intestinal representative of the *Verrucomicrobiota* (37). Qu et al. (38) observed a lower abundance of A. muciniphila in the feces of UC patients than in healthy controls. However, oral administration of the A. muciniphila markedly improved symptoms in acute colitis mice. Therefore, *Verrucomicrobiota* may provide potential prospects for treating human UC in the future.

### Association of metabolic phenotypes with anti-integrin therapy in UC patients.

Several studies revealed a significant relationship between metabolic profiles and clinical response in IBD patients underwent anti-TNF-α therapies (39–41). A previous study found that a disturbed lipid profile combined with apparent bile acid proved to predict the response to anti-TNF therapy (20). Another study identified predictive metabolites, specifically butyrate and substrates involved in its synthesis, for predicting clinical remission following biological therapy (39). The role of microbiota-derived metabolites in the clinical remission for UC patients receiving vedolizumab remains uncertain. Ananthakrishnan et al. (19) identified 13 significantly enriched pathways in baseline samples from CD patients commencing vedolizumab with remission; however, these researchers did not investigate SCFAs. Notably, we compared FFQs between remission and nonremission patients and found that these FFQs minimized the impact of food on metabolites. Our untargeted fecal metabolic profiling found significant differences in metabolic profiles, including increased levels of proline, scopolamine, salicyluric acid, riboflavin, quinolinic acid, and other metabolites between remission and nonremission groups in UC patients undergoing vedolizumab treatment. Furtherly, GC-MS analysis revealed significantly higher levels of butyric acid and isobutyric acid in patients with remission at baseline compared to the nonremission group, a finding consistent with Aden et al. (39). We also found significant correlations between butyric acid, propionic acid, valeric acid, and disease severity score (Mayo score), in line with Di’Narzo et al. (42).

### Longitudinal trajectory of the microbiome before and after treatment.

Prior studies have examined longitudinal changes in profile of gut microbiota of IBD patients before and after biological treatment. One recent study reported a lack of reduction in biodiversity and individual phylotypes among both remission and nonremission groups after anti-TNF therapy (39). Conversely, ameliorated fecal diversity was observed in responders to uncategorized treatment exposures in UC patients but not in CD patients (43). Moreover, gut diversity restoration has been reported in CD patients after anti-TNF treatment (44). Our study showed an increase in the genus *Lactobacillus* in UC patients who achieved remission after anti-integrin therapy at week 14. However, the nonremission group exhibited an increase in *Akkermansia* and a decrease in *Cetobacterium* and *Lactobacillus* at the genus level.

### USe of the gut microbiome and metabonomics pattern to predict clinical remission.

Shaw et al. (43) utilized baseline microbiome data and achieved 76.5% accuracy to predict the treatment response for IBD patients. Ding et al. (20) established high diagnostic value for fecal lipid signatures in predicting anti-TNF response (AUC = 0.94). In addition, a diagnosis model of microbial pathways was developed for CD patients starting anti-integrin therapy with moderate predictive accuracy (AUC = 0.738) (19). Lee et al. (45) developed a model using metagenomic, metabolomic, proteomic data in addition to clinical data, with the highest AUC value of 0.849. Notably, our study combined the fecal microbiota marker, butyrate, and isobutyric acid and achieved the highest discriminating ability with an AUR of 0.961 and a 95% CI of 0.882 to 1.000.

Our study has several shortcomings that must be acknowledged. First, the relatively small sample size of remission and nonremission patients from a single-center cohort may limit the statistical significance of our findings based on sequencing data sets. Second, the remission group was identified based on the Mayo score, which includes clinical indicators, biochemical, fecal, and endoscopic outcomes but not intestinal histology and pathology. Third, the follow-up time was limited due to numerous enrolled UC patients dropping out from long-term follow-up. A longer follow-up period of up to 1 year with fecal samples would have provided more robust evidence. Finally, several factors may influence the success or failure of anti-integrin therapy, including unknown genetic factors, drug secretion, and others. Despite these limitations, our study indicates the vital role of the gut microbial and metabolic milieu in anti-integrin therapy outcomes.

### Conclusions.

A significantly higher abundance of *Verrucomicrobiota* in patients with remission than in the nonremission group was found in the present study. In addition, the butyric acid and isobutyric acid levels were significantly higher in the remission group at baseline. A combination of *Verrucomicrobiota*, butyric acid, and isobutyric acid improved the diagnosis of early remission to anti-integrin therapy.

## MATERIALS AND METHODS

### Study protocol.

We conducted a single-center prospective cohort study. First, we evaluated the differences in the gut microbiome and metabonomics profiles between 11 healthy controls and 42 UC patients, including 13 inactive to mild UC patients and 29 moderate to severe UC patients who initiated anti-integrin therapy. Second, 29 patients with moderate to severe UC who initiated vedolizumab were monitored for 14 weeks. Subsequently, clinical and mucosal remission patients were divided into the remission group based on the Mayo score. The Mayo score includes four subscores: endoscopic findings, stool frequency, rectal bleeding, and physician global assessment. Each component is scored from 0 to 3. To predict remission to anti-integrin therapy, we thus evaluated the gut microbiome and metabonomics profile between remission and nonremission groups at baseline. The schematic illustration of this study is exhibited in Fig. S1.

### Patients.

All patients were recruited prospectively from December 2021 to January 2023 in the First Affiliated Hospital of USTC. Inclusion criteria included inpatients with moderate to severe UC in the setting of prior anti-TNF failure or naive to any biological therapy. Meanwhile, 11 subjects who had no history of gastrointestinal or metabolic disorders nor had used antibiotics or experienced medical treatment affecting gut transit or microbiota within the previous 6 months were recruited into the healthy control group. The following exclusion criteria were applied: pregnant women; patients who had abdominal surgery, fecal microbiota transplantation, or gastrointestinal malignancy; patients who were taking antibiotics or probiotics in the previous 4 weeks (that could impact intestinal flora); and patients whose clinical laboratory evidence indicated extraintestinal Clostridium difficile, cytomegalovirus, or Epstein-Barr virus infections. Participants with other comorbidities, including heart, lung, or cerebrovascular disease, irritable bowel syndrome, or an inability to undergo colonoscopy, were also ruled out. All participants completed a validated food frequency questionnaire (FFQ) to collect comprehensive dietary intake patterns (46). The self-administered FFQ about dietary information was monitored by two registered nutritionists (Q.Z. and Y.F.) and acquired the day before fecal samples collection, as reported in Table S1 in the supplemental material.

The study was approved by Ethics Committee of the First Affiliated Hospital of the USTC (no. 2023-KY065), and written informed consent was obtained before enrollment.

### Baseline disease activity assessment.

Two gastroenterologists (Y.Y. and H.F.) experienced in diagnosing IBD confirmed the presence of UC using medical history, endoscopy, histology, and radiographic techniques. The Mayo score was used to determine the UC disease activity. A total of 13 individuals were identified as being disease-free or showing mild disease activity, with a Mayo score of <6, while 29 patients commencing vedolizumab (anti-integrin) had an established diagnosis of moderate to severe disease activity, with a Mayo score of 6 to 12 and an endoscopic subscore of 2 or 3 (47). All UC patients underwent a laboratory measure of disease activity with erythrocyte sedimentation rate (ESR), fecal calprotectin (FC), and C-reactive protein (CRP), as well as biochemical parameters at baseline. Table S2 in the supplemental material contains the demographic information for all participants, whereas the objective assessment and biochemical features of the UC patients are summarized in Table S3.

### Clinical outcomes and follow-up.

The primary goal was clinical remission at week 14, also called early remission, for 29 patients starting anti-integrin therapy with vedolizumab. Therefore, the primary outcome was the difference in gut microbiome and metabonomics patterns between the early-remission and nonremission groups, whereas secondary outcomes were used to explore the difference in gut microbiome and metabonomics patterns among healthy controls, inactive to mild UC patients, and moderate to severe UC patients and to identify a continuous change in the microbiome with maintenance treatment.

The remission group was defined as a Mayo score of ≤2, with each subscore at ≤1, and a Mayo endoscopy subscore of ≤1 compared to the baseline (22). Patients who attained clinical remission were placed in the remission group, while the remaining patients were placed in the nonremission group. An inpatient service monitored all patients, and colonoscopy was performed to precisely obtain the Mayo score at baseline and week 14 after vedolizumab treatment by two gastroenterologists (N.H. and C.L.). Table 1 summarizes the clinical and biochemical evaluations between the remission and nonremission groups of moderate to severe UC patients. Figure 2 depicts the typical endoscopic appearance of remission and nonremission patients with pancolitis before and after vedolizumab treatment.

### Sample collection.

Fecal samples (*n* = 82) were collected from participants comprising healthy controls (*n* = 11), inactive to mild UC patients (*n* = 13), and UC patients with moderate to severe disease activity (*n* = 29 × 2, pre- and post-vedolizumab treatment). Aliquots of samples containing 2 g of feces were immediately frozen at −20°C and later transported to a −80°C freezer within 24 h for storage before assessing and analyzing the fecal microbiota and metabolites (48, 49).

### Use of 16S rRNA sequencing to analyze fecal microbiota.

Sterile collecting spoons were used to collect fresh fecal samples from all participants. Each sample was poured into a 3-mL preservation solution and stored at −80°C. 16S rRNA sequencing was carried out to detect the gut microbiota. A hexadecyltrimethylammonium bromide/sodium dodecyl sulfate (CTAB/SDS) approach was performed to extract DNA from samples. The extracted DNA was diluted to 1 μg/μL of sterile water to monitor the DNA concentration and purity. The primers used to amplify the 16S rRNA of V3-V4 regions were 341F (5′-CCTACGGGNGGCWGCAG-3′) and 805R (5′-GACTACHVGGGTATCTAATCC-3′) with the barcode. PCRs were performed using 15 μL of Phusion High-Fidelity PCR Master Mix (New England Biolabs). The PCR products were purified using a gel extraction kit (Qiagen, Hilden, Germany) after merging was performed in equidensity ratios. Subsequently, sequencing libraries were provided by using a TruSeq DNA PCR-Free sample preparation kit (Illumina, USA) and sequenced on an Illumina NovaSeq platform, generating 250-bp paired-end reads. Paired-end reads were assigned to samples and merged by applying FLASH (vI.2.7) (50). Quality filtering of the raw tags was conducted to gain the high-quality clean tag on QIIME (v1.9.1) (51). The UCHIME algorithm was used to compare tags to the Silva database to eliminate chimera sequences (52, 53). UPARSE (v7.0.1001) was used to assign identical operational taxonomic units (OTUs) to sequences with (>97%) similarity. Using the Silva Database, the Mothur algorithm was applied to annotate taxonomic information for each representative sequence (54). The figure visualization methods employed here to detect differences in diversity between groups were PCA and PCoA to measure the β-diversity and Shannon’s index to measure the α-diversity. The software used for analysis was QIIME (v1.9.1). NMDS was used as a nonlinear model to identify cluster differences according to the Bray-Curtis distance applying the R software (v3.5.2) with the vegan package. Differential OTUs between groups were recognized using the DEseq2 package, with a significance threshold set at *P* < 0.05, where log_2_(fc) > |1|.

### Untargeted fecal metabolomic analysis.

The liquid chromatography-tandem mass spectrometry (LC-MS/MS) method was used to analyze the untargeted metabolomic patterns of fecal samples (55). UHPLC system (Vanquish; Thermo Fisher Scientific) coupled with a UPLC BEH Amide column (2.1 mm × 100 mm, 1.7 μm) to a QExactive HFX mass spectrometer (Orbitrap MS; Thermo). The data cleaning, statistical analysis, and pathway enrichment analysis were performed using MetaboAnalyst (https://www.metaboanalyst.ca). The figures were calculated separately for positive and negative ions. Samples with a peak intensity matrix of zero values in more than half of the samples were filtered by eliminating peaks. Subsequently, the remaining missing values were changed to one-fifth of the minimum positive value for each variable. The mean value was used to normalize the variables with a relative standard deviation of deviating values (>25%). Significant differences in untargeted fecal metabolites between groups were detected utilizing the “orthogonal projections to a latent structures discriminant analysis” (OPLS-DA) algorithm. The robustness of the OPLS-DA model was tested using a permutation test (100 permutations). The differentially expressed metabolites (DEMs) were confirmed based on the criteria of variable importance in the projection value of >1, where |log_2_(fc)| > 2 and *P* < 0.05. Meanwhile, MetaboAnalyst was used to perform DEM-related Kyoto Encyclopedia of Genes and Genomes (KEGG) pathways enrichment analysis, and underlying targets were displayed with *P* < 0.05.

### Measurement of fecal short-chain fatty acids.

One gram of fresh fecal sample was collected in a sterile collecting spoon from each UC patient receiving vedolizumab. Total fecal DNA was extracted by using a QIAamp DNA stool minikit (Qiagen). The collected samples were analyzed for fecal SCFA content within 24 h of collection. A previously described GC-MS method was used to evaluate fecal SCFA content (56, 57). One gram of crude feces was subjected to lyophilization and reweighed after lyophilization. The fecal SCFA concentrations were determined by utilizing the sample weight after lyophilization, which was expressed in micrograms per grams. The lyophilized fecal samples were dissolved in 5 mL of 0.5% phosphoric acid solution; 240 μL of this solution was used to dilute 60 μL of the supernatant. The resultant solution underwent sonication for 5 min and centrifugation at 3,000 relative centrifugal force for 10 min. The Geno/Grinder 2010 (SPEX, Metuchen, NJ) was used to extract SCFAs from the sample using 300 μL of butanol. Before GC-MS analysis, isotopically labeled internal standards were run on an Agilent 7890A gas chromatograph equipped with a MultiPurpose Sampler MPS (GERSTEL, Mülheim an der Ruhr, Germany) and a Pegasus GC-TOFMS system (Leco Corporation, St. Joseph, MI). A polar VFWAXms capillary column (30 m × 0.25 mm [inner diameter] × 0.25 μm film thickness; Agilent Technologies, Santa Clara, CA) was employed for separation purposes. The separation was carried out under a helium carrier gas flow rate of 1 mL/min. The split mode was injected by 1 μL of the sample at a 1:10 ratio. The oven temperature was started at 70°C for 1 min and gradually increased, ultimately being maintained at 240°C for 2 min, with a total run time of 15.8 min. The data were collected in full scan mode, using a mass range of *m/z* 40 to 550 with an electron impact ionization of 70 eV.

### Statistical analysis.

Categorical data were presented as numbers and percentages. Normally distributed data are expressed as means ± the standard deviations, while non-normally distributed data are expressed as medians with their corresponding interquartile ranges. A chi-square test was employed to analyze categorical data, while a Fisher exact test was employed in cases where the field frequency was <5. Normally distributed continuous and nonparametric data between two groups were compared using a the two-tailed Student *t* test and a Mann-Whitney U test, respectively. One-way analysis of variance was used to analyze multiple parametric groups, while a Kruskal-Wallis H test was employed to compare the distribution of nonnormal data among the three groups. Spearman’s correlation highlighted the probable relationship between fecal SCFAs and symptom severity (total Mayo score) in UC patients. The receiver operating characteristic (ROC) with the area under the curve (AUC) was implemented to measure the diagnostic ability of the model to evaluate the gut microbiome performance and metabonomics profiles to differentiate between the vedolizumab remission and nonremission group. Logistic regression models were used to study effects of predictor variables. The difference was considered statistically significant when the *P* value was <0.05.

### Data availability.

The raw sequence data have been deposited in the SRA database under accession number PRJNA953829. Additional data used to support the findings of this study are available from the corresponding author upon request.
