# Supplementary figures and images for: Gut Microbiome and Metabonomic Profile Predict Early Remission to Anti-Integrin Therapy in Patients with Moderate to Severe Ulcerative Colitis
Source: Microbiol Spectr. 2023 May 18;11(3):e01457-23. doi: 10.1128/spectrum.01457-23 (PMC10269848; doi:10.1128/spectrum.01457-23)

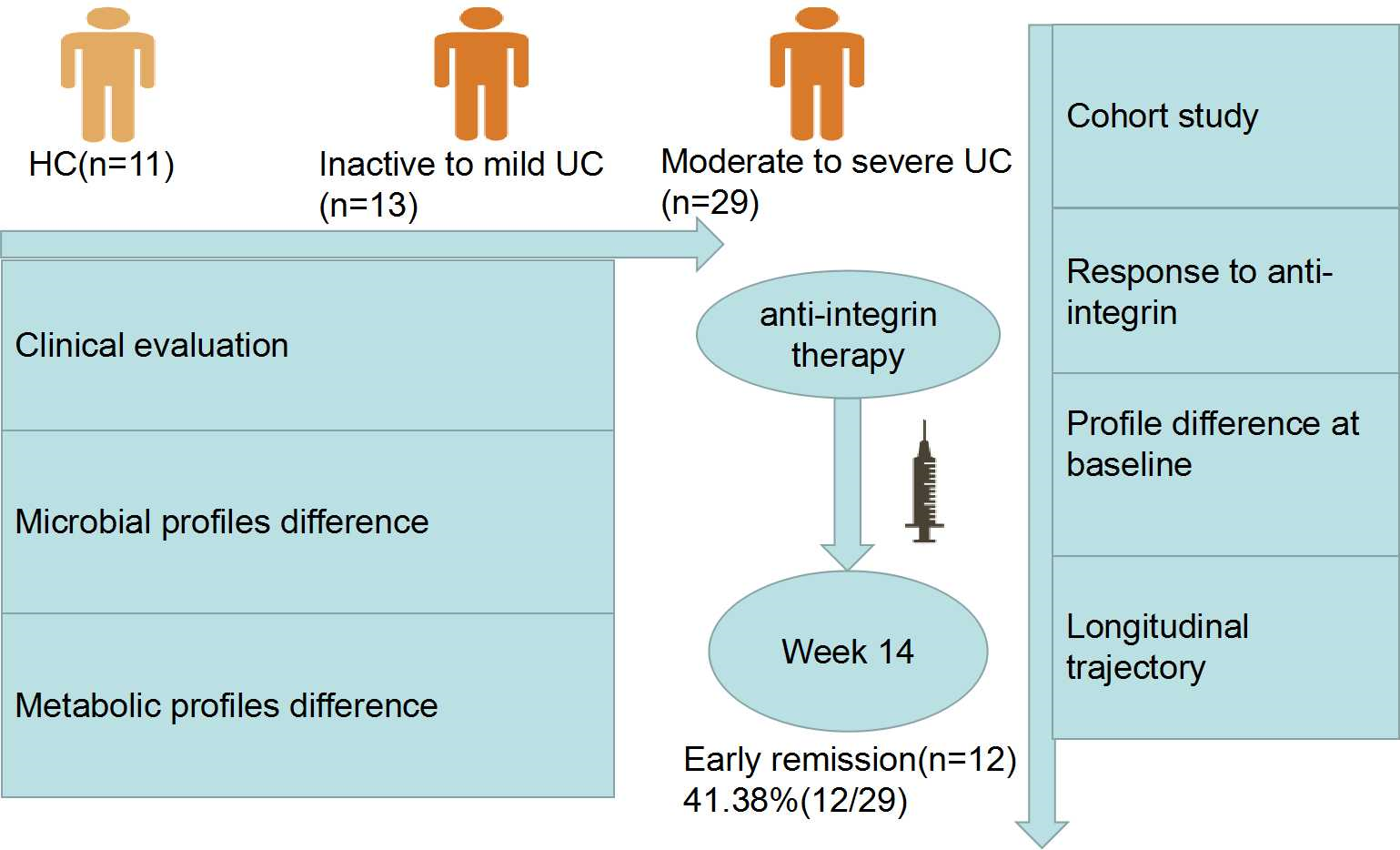

Supplement: Supplemental file 1 — Figure S1. Download spectrum.01457-23-s0001.tif, TIF file, 0.8 MB [file spectrum.01457-23-s0001.tif]

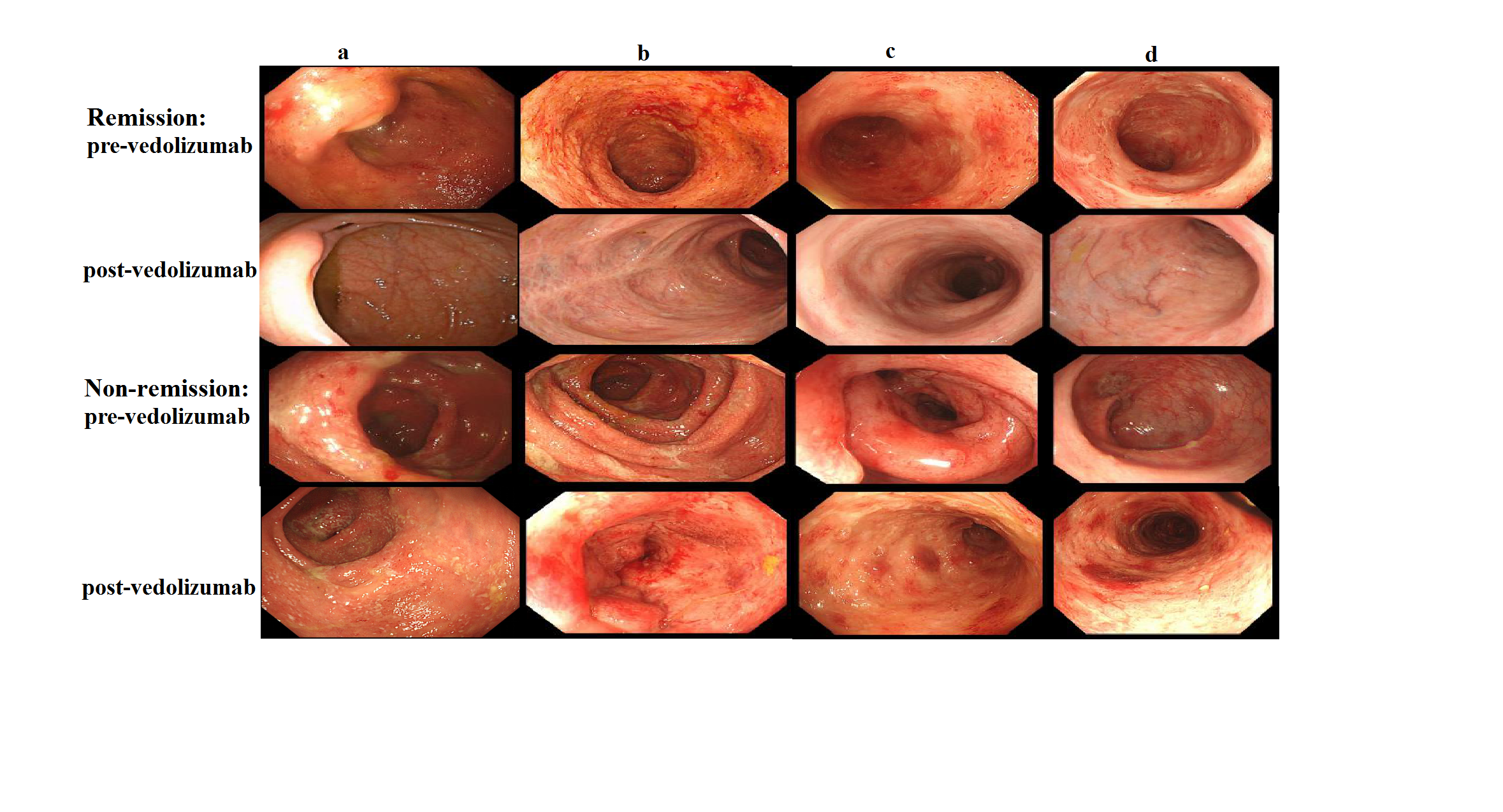

Supplement: Supplemental file 2 — Figure S2. Download spectrum.01457-23-s0002.tif, TIF file, 2.2 MB [file spectrum.01457-23-s0002.tif]

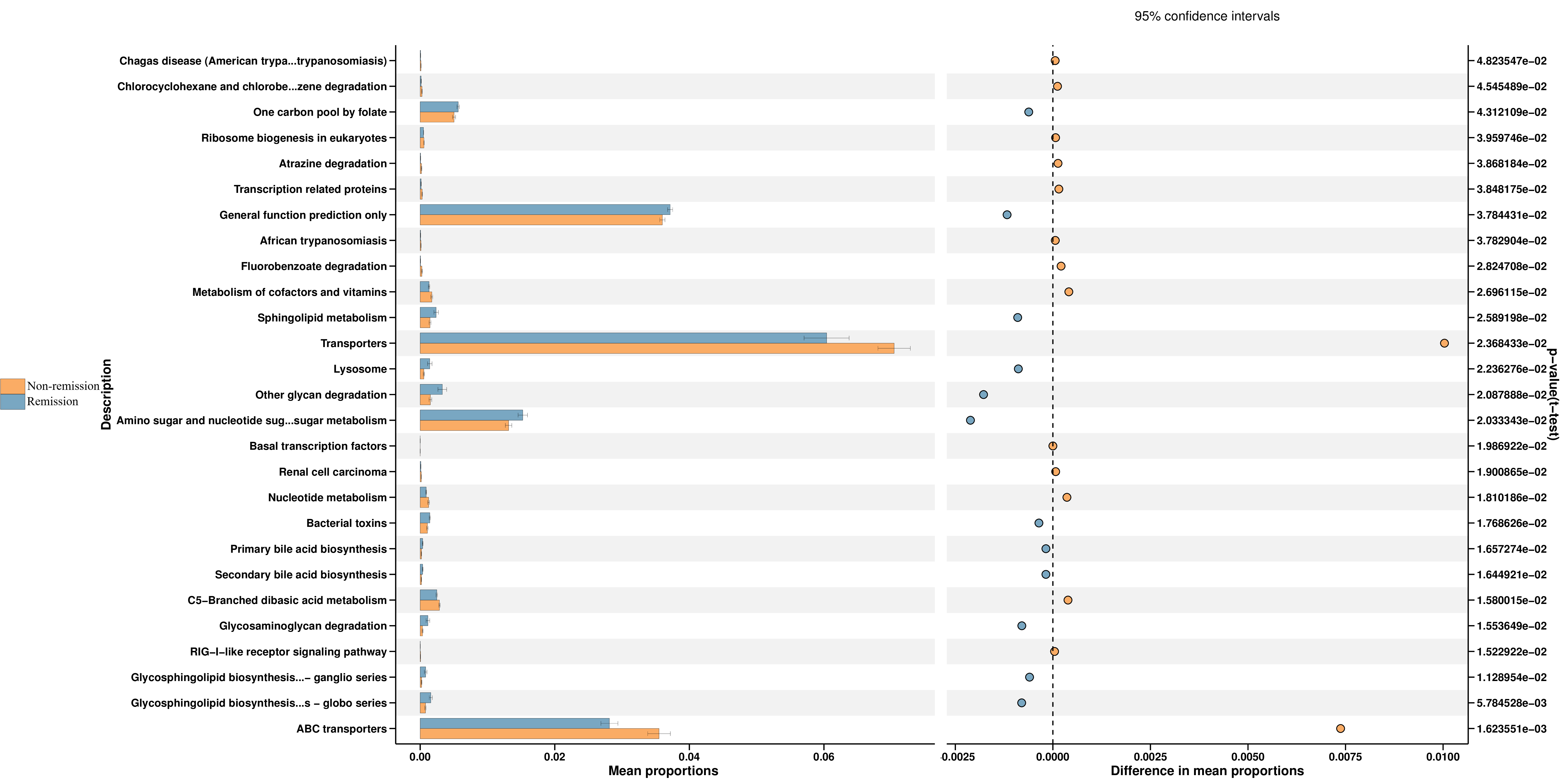

Supplement: Supplemental file 3 — Figure S3. Download spectrum.01457-23-s0003.tif, TIF file, 1.2 MB [file spectrum.01457-23-s0003.tif]

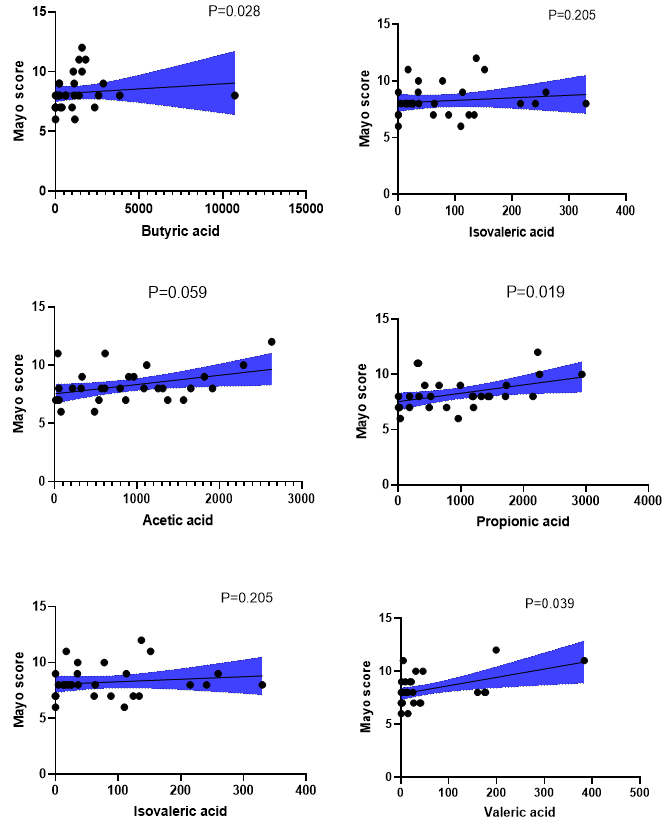

Supplement: Supplemental file 4 — Figure S4. Download spectrum.01457-23-s0004.tif, TIF file, 0.08 MB [file spectrum.01457-23-s0004.tif]
