# Supplementary material for: Gut Microbiome and Metabonomic Profile Predict Early Remission to Anti-Integrin Therapy in Patients with Moderate to Severe Ulcerative Colitis
Source: Microbiol Spectr. 2023 May 18;11(3):e01457-23. doi: 10.1128/spectrum.01457-23 (PMC10269848; doi:10.1128/spectrum.01457-23)
Supplement: Supplemental file 5 — Legends to Fig. S1 to S4. Download spectrum.01457-23-s0005.docx, DOCX file, 0.01 MB [file spectrum.01457-23-s0005.docx]

**Supplementary Figure 1.** The graphical abstract of this study.

**Supplementary Figure 2.** An endoscopic appearance of pancolitis pre- and post-vedolizumab treatment between remission and non-remission groups after 14 weeks follow-up in moderate to severe UC patients. The endoscopic appearance includes mucosal hyperemia and edema, mucous exudate erosions, multiple ulcers, and spontaneous bleeding in the (a) ileocecum, (b) ascending colon, (c) sigmoid colon, and (d) rectum before and after anti-integrin therapy.

**Supplementary Figure 3.** The difference in the direction of change for pathways between UC patients who achieved remission and those who did not. The functional prediction analysis showed the potential pathways, including the superpathway of L-threonine biosynthesis; pyruvate fermentation to propanoate; cis-vaccenate biosynthesis, and others.

**Supplementary Figure 4.** The relationship between SCFAs and disease severity score (Mayo score). Spearman's correlation analysis revealed significant associations between disease severity score (Mayo score) and propionic acid (P = 0.019), butyric acid (P = 0.028), and valeric acid (P = 0.039) in moderate to severe UC patients.
