# Supplementary material for: Gut Microbiome and Metabonomic Profile Predict Early Remission to Anti-Integrin Therapy in Patients with Moderate to Severe Ulcerative Colitis
Source: Microbiol Spectr. 2023 May 18;11(3):e01457-23. doi: 10.1128/spectrum.01457-23 (PMC10269848; doi:10.1128/spectrum.01457-23)
Supplement: Supplemental file 6 — Tables S1 to S4. Download spectrum.01457-23-s0006.docx, DOCX file, 0.02 MB [file spectrum.01457-23-s0006.docx]

| Food and nutrients | Healthy controls (n=11) | Inactive to mild UC patients (n=13) | Moderate to severe UC(n=29) | | #p value | *p value |
| --- | --- | --- | --- | --- | --- | --- |
|  |  |  | responders(n=12) | Non-responders(n=17) |  |  |
| Meat, servings | 1.0(0.5,2.0) | 1.0(0.5,1.5) | 1.0(1.0,2.0) | 1.0(0.5,2.0) | 0.866 | 0.347 |
| Fish, servings | 0.5(0.5,1.0) | 0.5(0.5,1.0) | 0.5(0.5,1.0) | 0.7(0.3,1.0) | 0.683 | 0.983 |
| Eggs, servings | 1.0(0.5,1.0) | 1.0(0.2,1.0) | 1.0(1.0,1.0) | 1.0(0.3,1.0) | 0.256 | 0.556 |
| Soy, servings | 0.5(0.3,1.0) | 0.5(0.0,1.0) | 0.5(0.3,1.0) | 0.5(0.3,1.0) | 0.867 | 0.948 |
| Vegetables, servings | 1.0(1.0,1.0) | 1.0(1.0,1.0) | 1.0(1.0,2.0) | 1.0(1.0,1.0) | 0.690 | 0.879 |
| Fruits, servings | 1.0(0.5,1.0) | 0.5(0.3,1.0) | 1.0(1.0,1.0) | 1.0(0.3,1.0) | 0.575 | 0.370 |
| Nuts, servings | 0.0(0.0,0.0) | 0.0(0.0,0.0) | 0.0(0.0,0.0) | 0.0(0.0,0.0) | 0.982 | 0.913 |
| Energy, kcal | 1915.0(1760.0,2075.0) | 1885.0(1561.5,2015.0) | 1721.5(1617.5,2015.0) | 1815.0(1561.5,1924.0) | 0.443 | 0.845 |
| **Protein (% energy)** |  |  |  |  |  |  |
| Animal protein, g | 15.0(11.0,25.0) | 14.0(11.0,24.0) | 18.0(12.0,26.0) | 16.0(13.0,28.0) | 0.984 | 0.283 |
| Plant protein, g | 29.0(25.0,40.0) | 32.0(27.0,45.0) | 30.0(26.0,40.0) | 31.0(27.0,39.0) | 0.163 | 0.471 |
| **Fat (% energy)** |  |  |  |  |  |  |
| Saturate fat, g | 10.0(8.0,16.0) | 11.0(8.0,18.0) | 9.0(6.0,17.0) | 12.0(7.0,15.0) | 0.984 | 0.811 |
| Monounsaturated fat, g | 19.0(18.0,25.0) | 18.0(12.0,25.0) | 20.0(13.0,24.0) | 21.0(14.0,26.0) | 0.129 | 0.913 |
| Polyunsaturatd fat, g | 11.0(7.0,17.0) | 12.0(8.0,19.0) | 10.0(8.0,15.0) | 10.0(6.5,16.0) | 0.857 | 0.913 |
| **Carbohydrate (% energy)** |  |  |  |  |  |  |
| **Dietary fiber, g** | 19.0(16.0,25.0) | 19.0(14.0,26.0) | 20.0(15.0,28.0) | 21.0(17.0,27.0) | 0.723 | 0.195 |

Supplemental Table 1. Dietary intakes of patients with UC and normal controls based on Food Frequency Questionnaires in the current study.

Abbreviations: IQR = interquartile range;

A serving of meat, fish, eggs, soy is defined as 7g protein; a serving of dairy is defined as 8g protein; a serving of vegetable is defined as 100g; a serving of fruit is defined as 15g carbohydrates; a serving of nuts is defined as 45 kcal.

The difference among healthy controls, inactive to mild UC and moderate to severe UC groups represented by #p value was analyzed using Kruskal-Wallis H-test.

The difference between responder and non-responder groups represented by *p value was analyzed using Mann-Whitney U-test.

Supplementary Table 2. Summary of demographics for the three groups.

| Parameters | Healthy controls(n=11) | Inactive to mild UC(n=13) | Moderate to severe UC(n=29) | p value |
| --- | --- | --- | --- | --- |
| Age(years) | 36.09±7.57 | 46.00±15.72 | 42.76±15.67 | 0.242 |
| Female(%) | 5 | 6 | 10 | 0.702 |
| Duration(months) | / | 56.46±19.15 | 66.52±18.20 | 0.969 |
| BMI(kg/m^2^) | 21.21±1.24 | 20.79±2.30 | 21.73±2.32 | 0.400 |

The difference among healthy controls, inactive to mild UC and moderate to severe UC groups was analyzed using ANOVA test.

The difference between inactive to mild UC and moderate to severe UC groups was analyzed using t test.

Supplementary Table 3. Summary of clinical and biochemical characteristics of UC patients.

| Parameters | Inactive to mild UC(n=13) | Moderate to severe UC(n=29) | t | p value |
| --- | --- | --- | --- | --- |
| Haemoglobin (g/L) | 120.69±18.26 | 118.10±21.91 | -0.371 | 0.712 |
| White cell count (x10 9 /L) | 5.53±1.37 | 6.84±2.19 | 1.993 | 0.053 |
| Platelet count (x10 9 /L) | 275.85±103.20 | 245.38±79.60 | -1.045 | 0.302 |
| ESR (mm/hr) | 10.38±3.53 | 33.03±11.04 | 2.589 | 0.013 |
| **Inflammatory biomarkers** |  |  |  |  |
| C-reactive protein (mg/L) | 8.97±4.19 | 18.09±5.23 | 2.138 | 0.039 |
| Calprotectin (μg/g) | 54.46±26.73 | 142.10±36.01 | 2.226 | 0.032 |
| **Liver function** |  |  |  |  |
| Bilirubin (μM) | 11.38±5.18 | 12.14±5.07 | 0.447 | 0.658 |
| Alkaline phosphatase (IU/L) | 83.38±28.45 | 82.45±46.38 | -0.067 | 0.947 |
| Alanine aminotransferase (IU/L) | 15.96±9.21 | 16.30±14.88 | 0.075 | 0.941 |
| Aspartate aminotransferase(IU/L) | 17.78±5.15 | 18.80±8.07 | 0.419 | 0.678 |
| Albumin (g/L) | 37.43±4.57 | 37.65±5.25 | 0.133 | 0.895 |
| **Renal function** |  |  |  |  |
| Creatinine (μM) | 57.85±12.52 | 64.10±13.06 | 1.453 | 0.154 |
| **MAYO score** | 3.77±0.83 | 8.24±1.46 | 10.305 | <0.001 |

Supplementary Table 4. The correlations between fecal SCFAs and symptom severity in UC patients receiving vedolizumab at baseline.

| Fecal level of SCFAs | **MAYO score** | |
| --- | --- | --- |
|  | Spearman ρ | p value |
| Acetic acid (C2) | 0.355 | 0.059 |
| Propionic acid (C3) | 0.435 | 0.019 |
| Butyric acid (C4) | 0.408 | 0.028 |
| Isobutyric acid (iC4) | 0.203 | 0.292 |
| Isovaleric acid (iC5) | 0.242 | 0.205 |
| Valeric acid (C5) | 0.385 | 0.039 |

Spearman’s rank sum tests were applied to evaluate the correlations between SCFAs and disease severity.
